# Relative quantification of *PIK3CA *gene expression level in fine-needle aspiration biopsy thyroid specimens collected from patients with papillary thyroid carcinoma and non-toxic goitre by real-time RT-PCR

**DOI:** 10.1186/1756-6614-3-5

**Published:** 2010-08-30

**Authors:** Katarzyna Wojciechowska-Durczyńska, Kinga Krawczyk-Rusiecka, Anna Cyniak-Magierska, Arkadiusz Zygmunt, Elżbieta Gałecka, Andrzej Lewiński

**Affiliations:** 1 Department of Endocrinology and Metabolic Diseases, Medical University of Lodz, Polish Mother's Memorial Hospital - Research Institute, Lodz, Poland

## Abstract

**Background:**

Recent studies have shown that the phosphatidylinositol 3-kinase (PI3K) signaling pathway is important regulator of many cellular events, including apoptosis, proliferation and motility. PI3K pathway alterations (*PIK3CA *gene mutations and/or amplification) have been observed in various human tumours. In the majority of diagnosed cases, mutations are localized in one of the three "hot spots" in the gene, responsible for coding catalytic subunit α of class I PI3K (*PIK3CA*). Mutations and amplification of *PIK3CA *gene are characteristic for thyroid cancer, as well.

**Methods:**

The aim of our study was to examine a gene expression level of *PIK3CA *in fine-needle aspiration biopsy (FNAB) thyroid specimens in two types of thyroid lesions, papillary thyroid carcinoma (PTC) and non-toxic goitre (NTG). Following conventional cytological examination, 42 thyroid FNAB specimens, received from patients with PTC (n = 20) and NTG (n = 22), were quantitatively evaluated regarding *PIK3CA *expression level by real-time PCR in the ABI PRISM^® ^7500 Sequence Detection System.

**Results:**

Significantly higher expression level (RQ) of *PIK3CA *in PTC group has been noted in comparison with NTG group (p < 0.05).

**Conclusion:**

These observations may suggest role of *PIK3CA *alterations in PTC carcinogenesis.

## Background

Phosphatidylinositol 3-kinase (phosphoinositide 3-kinase, PI3K) family is composed of three classes of kinases. Class I PI3K regulates cell growth, proliferation and survival. It consists of two subclasses: Ia - dimeric components, comprised of catalytic subunits: p110α, p110β, p110δ, associated with p85 regulatory subunit and subclass Ib, which is heterodimer consisting of p110γ catalytic subunit, connected with p101 regulatory subunit. Subclass Ia is responsible for transmission signals from receptor tyrosine kinase - RTK (i.e. EGFR, PDGFR). Regulation of this signal transduction pathway is frequently disrupted in human cancers by alterations of PI3K pathway. Among the four isoforms of the catalytic subunits, the α-type has been shown to harbour oncogenic mutations or amplifications in its gene (*PIK3CA*) [1,2]. *PIK3CA *gene is located on chromosome 3q26.3 and consists of 20 exons, coding 1068 amino acids and yielding a 124 kDa size protein [2]. Eighty percent of the mutations in *PIK3CA *gene are located in three regions within the helical (exon 9), catalytic (exon 20) and p85 binding (exon 1 and 2) domains [3]. The location of the mutations induces one of the several distinct mechanisms [4], enhancing PIK3CA kinase activity and Akt phosphorylation.

The highest frequency of activating mutations of *PIK3CA *gene has particularly been found in breast [5] and colorectal [3] cancers. The incidence rates of these mutations indicate that *PIK3CA *is one out of the two most commonly mutated genes, identified in human cancers (the other one being *KRAS*). In addition to activating mutations, *PIK3CA *gene amplification has also been implicated in cancer and has been associated with increased PI3K signaling, for instance, in ovarian [6] and cervical [7] cancers. The increased copy number is associated with increased cell growth and decreased apoptosis. The above mentioned results of numerous studies have established the role of *PIK3CA *as an oncogene in human cancers. PI3K is also thought to be involved in the formation of metastatic phenotype and promotion of carcinoma invasion. This phenotype appears to be independent of kinase Akt activation.

Over the past several years, the frequency of *PIK3CA *mutations and gene amplification has been analyzed in some sporadic thyroid carcinomas [8-12]. Similarly to other human cancers, the majority of *PIK3CA *gene mutations in thyroid neoplasms has been reported in exon 9 (helical domain) and 20 (kinase domain) [10]. Mutations in *PIK3CA *gene have been observed more frequently in follicular thyroid carcinoma (FTC) and anaplastic thyroid carcinoma (ATC), while being rather uncommon in PTC [8,10,11]. Still, little is known about the precise role of *PIK3CA *in PTC and data are not consistent.

In the present paper, we have evaluated the relative expression level of *PIK3CA *gene in PTC and NTG. Additionally, we attempted to examine a potential correlation between *PIK3CA *gene expression level in FNAB specimens of thyroid tissue and age and gender of patients with PTC and NTG. To our knowledge, this is - up to-date - the first report which provides data on these subjects.

## Methods

The patients were selected on the basis of the results of routinely performed FNAB of the thyroid gland. Cytological specimens from 42 patients (35 women, 7 men) were examined, including 20 patients with PTC, and 22 patients with NTG. All tissue samples were taken after patients' informed consents were obtained. PTC and NTG were diagnosed, according to cytological evaluation of FNAB specimens. Each aspirate was smeared for routine cytology, while the remaining part of aspirate was immediately washed out of the needle. The cells, obtained from the needle, were used in further investigation. Macroscopically unchanged thyroid tissue, surgically removed from patients with thyroid cancer, served as a control for *real-time *PCR experiment.

Total RNA from FNAB was extracted by use of an RNeasy Micro Kit (Qiagen, Hilden, Germany), based on modified Chomczyński and Sacchi's method, according to manufacturer's recommendations. The purity of total RNA was assessed by NanoDrop^® ^ND-100 spectrophotometr (data not presented). One hundred nanogram of total RNA was used in the first strand cDNA synthesis with TaqMan^® ^Reverse Transcripton Reagents (Applied Biosystems, Branchburg, New Jersey, USA), according to manufacturer's instruction.

Real-time PCR was performed on the ABI PRISM^® ^7500 Sequence Detection System (Applied Biosystem, Foster City, CA, USA), by using *Taq*Man^® ^Universal PCR Master Mix (Applied Biosystem) and *Taq*Man^® ^Gene Expression Assays probe and primer mix (Applied Biosystem), according to the manufacturer's specification. The Assay Identification number of *PIK3CA *was: Hs00180679_ml. Gene-specific probes were labeled by using reporter dye FAM at the 5'end. A non-fluorescent quencher and the minor grove binder were linked at the 3'end of probe. Thermal cycler conditions were as follows: hold for 10 min. at 95°C, follow by two-step PCR for 50 cycles of 95°C for 15 s, followed by 60°C for 1 min. Amplification reactions in triplicate for each sample were performed and the results were normalized to the *ACTB *gene expression level.

An analysis of relative gene expression data was performed, using the 2^-ΔΔCT ^method on an ABI PRISM^® ^7500 Sequence Detection System Software (Applied Biosystems, Foster City, CA, USA). The fold change in studied gene expression, normalised to endogenous control, was calculated using: RQ = 2^-ΔΔCT ^(Table 1 and Table 2).

**Table 1:** Gender, age, ddCT and RQ values in PTC group

**No**.	Gender	Age	ddCT values	RQ values
1.	M	58	-7.105	137.671

2.	M	64	-3.288	9.77

3.	F	49	-2.239	4.721

4.	F	67	-5.185	36.378

5.	F	28	-0.307	1.237

6.	F	56	-7.224	149.519

7.	F	57	-3.675	12.771

8.	F	39	-2.968	7.827

9.	F	36	-4.017	16.187

10.	F	49	-4.2	18.373

11.	F	28	-1.837	3.572

12.	F	19	-4.923	30.343

13.	M	61	-1.418	2.672

14.	F	28	0.214	0.862

15.	F	50	-0.689	1.612

16.	F	53	-1.789	3.455

17.	F	no data	-2.757	6.758

18.	F	52	-4.985	31.676

19.	F	24	-3.735	13.311

20.	M	56	-3.94	15.345

**Table 2:** Gender, age, ddCT and RQ values in NTG group

**No**.	Gender	Age	ddCT values	RQ values
1.	F	77	-5.493	45.046

2.	F	27	-2.853	7.223

3.	F	68	-2.268	4.817

4.	F	62	-0.844	1.795

5.	F	43	-1.852	3.609

6.	F	71	8.186	0.03

7.	F	43	-3.068	8.386

8.	F	49	-0.851	1.804

9.	M	61	-4.473	22.213

10.	M	44	8.492	0.03

11.	F	79	7.237	0.007

12.	F	54	5.855	0.009

13.	F	50	8.704	0.002

14.	F	51	-1.478	2.785

15.	M	55	-1.362	2.571

16.	F	41	-0.097	1.069

17.	F	68	-3.158	8.927

18.	F	87	-2.334	5.046

19.	F	62	0.291	0.813

20.	F	64	-3.88	14.719

21.	F	43	-1.252	2.382

22.	F	70	-4.115	17.323

### Statistical analysis

Analysis was performed with statistical software package Statistica 7. Basic measures of location (i.e. mean, median), measures of dispersion (SD, SEM) and minimum and maximum and lower quartile and upper quartile values were calculated to provide detailed descriptions of gene expressions in selected groups. Subsequently, the data were statistically analyzed, using non-parametric Mann-Whitney's *U *test, in order to compare the level of expression values (RQ) among the two studied independent groups (PTC, NTG). Spearman's rank correlation coefficient was used to describe the correlation between *PIK3CA *gene expression level and patient's age in studied groups. Statistical evaluations of correlation between the gene expressions and gender were performed using the non-parametric Mann-Whitney's *U*-test.

## Results

**Figure 1:**
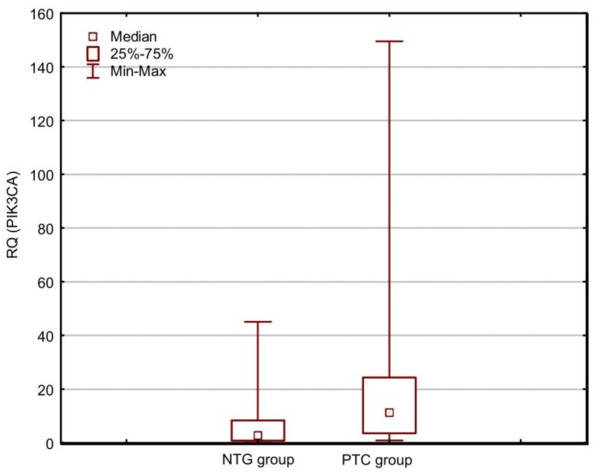
**Box-and-whisker plots, representing the expression of *PIK3CA *gene in the studied groups (PTC, NTG)**. The results are calculated as RQ values. Whiskers represent median and minimum and maximum values for particular groups. Boxes represent lower quartile and upper quartile. The results were statistically analyzed, using Man-Whitney's *U *test (p < 0.05).

**Figure 2:**
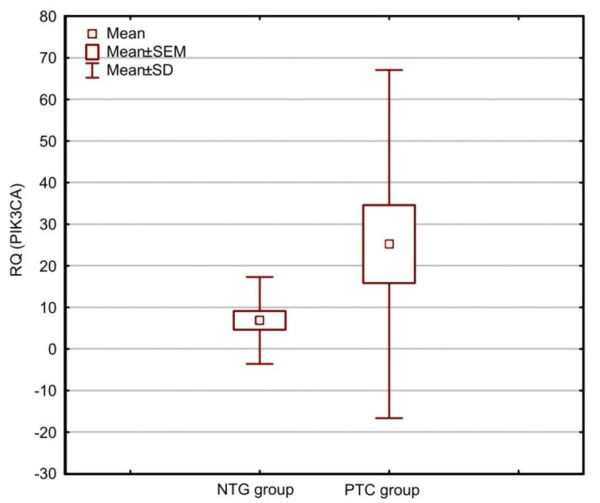
**Box-and-whisker plots, representing the expression of *PIK3CA *gene in the studied groups (PTC, NTG)**. The results are calculated as RQ values. Whiskers represent means ± SD (standard deviation) for particular groups. Boxes represent means ± SEM (standard error of mean). The results were statistically analyzed, using Man-Whitney's *U *test (p < 0.05).

Our analysis (Mann-Whitney's *U*-test), used for RQ comparison, revealed statistically significant (p < 0,05) differences in *PIK3CA *gene expression levels between PTC and NTG. The expression level (RQ) of the *PIK3CA *was higher in PTC group, in comparison with NTG group. There was no correlation with patient's sex and age (p > 0.05) (Spearman's rank correlation test and Mann-Whitney's *U*-test). The box-and-whisker plot diagrams, representing the different expression levels of *PIK3CA *(median and mean values) in PTC and NTG group, are shown in Figure 1 and Figure 2, respectively.

## Discussion

In our study, we have clearly presented significantly higher *PIK3CA *gene expression in FNAB-derived samples of PTC, in comparison with NTG group. Thus, overexpression of *PIK3CA *gene within PTC can be regarded as a potential genetic event in thyroid carcinogenesis. Our results are consistent with data published by Abubaker et al. [12] who have presented the high incidence of *PIK3CA *amplification in PTC among the Middle East population. *PIK3CA *amplifications and mutations (exon 9 and 20) were discovered in 53.1% and 2% of PTC group, respectively [12].

So far, insulin-like growth factor I (IGF-I) and transforming growth factor α (TGF-α) receptors with tyrosine kinase activity have been proved to commonly be overexpressed in thyroid neoplasms. Furthermore, autoendocrine effects of IGF-I and TGF-α have been noticed, suggesting their association with cancer progression. The increased signaling in these receptors, mediating thyroid cancer development, activates PI3K pathway which is a major intracellular downstream mediator [13]. It may explain the increased activation of PI3K pathway in thyroid malignancies, documented in our study.

Moreover, genetic alterations of *PIK3CA *gene have been proved to increase expression of *PIK3CA *gene, as well. Amplifications and mutations in *PIK3CA *gene have been reported in many human cancer types, including thyroid cancer. To date, the increased activity of PI3K pathway was noted particularly in sporadic thyroid carcinomas, especially in FTC and ATC. Previous studies have revealed higher prevalence of *PIK3CA *copy gain in ATC (42%) in comparison with FTC (28%), which may indicate that *PIK3CA *gene participates in the progression of FTC toward more aggressive types of cancer [10,14]. Furthermore, available data indicate that the occurrence rate of *PIK3CA *mutations in PTC is low and the rate in question is reported to be in range from 0 to 3% [8-12]. Gene amplification of *PIK3CA *is more common (ranges from 5 to 14%) in PTC patients, however, still it is less frequent in comparison with FTCs [8-12]. These findings support the hypothesis that *PIK3CA *is essential for PTC carcinogenesis and for an invasive type of cancer development. Supporting these observations, Wu et al. [9] found a strong association of *PIK3CA *copy gain with high-risk clinico-pathological features in thyroid cancer; the authors suggested a possible role of that amplification in thyroid tumor progression.

However, basing on the well-defined role for BRAF and ERK signaling, *PIK3CA *alterations are rather unlikely to play alone a central role in PTC carcinogenesis [14]. Far more possible, *PIK3CA *genetic changes may coparticipate in multi-step model of carcinogenesis pathway, likewise in the colorectal cancer, though these interactions require further evaluation.

On the other hand, mutant PI3K may be an important goal for future targeted tumor therapy. *PIK3CA *mutations enhance PI3K function, which - contrary to function loss - is much easier to control. Still, mostly enigmatic molecular pathomechanism of PIK3CA activity increase remains the greatest barrier to obtain highly specific and effective targeted therapy. This task requires further investigations, according to results of clinical studies [15].

## Conclusions

In conclusion, our results on overexpression of *PIK3CA *in PTC, suggest that this gene is involved, to some degree, in pathogenesis of PTC, likewise in more aggressive types of the cancer in question. The definite role of *PIK3CA *in multi-step carcinogenesis and acquisition of invasive capacity of thyroid cancers remains to be determined.

## Competing interests

The authors declare that they have no competing interests.

## Authors' contributions

KW-D designed and coordinated the study, carried out the molecular genetic studies and drafted the manuscript. KR-K participated in performing molecular studies. AC-M participated in performing molecular studies. AZ participated in coordination of the study. EG participated in coordination of the study. AL, the senior author, wrote the manuscript. All authors have read and approved the final manuscript.
